# In Vivo and In Vitro Evaluation of the Protective Effects of Hesperidin in Lipopolysaccharide-Induced Inflammation and Cytotoxicity of Cell

**DOI:** 10.3390/molecules25030478

**Published:** 2020-01-22

**Authors:** Rasha Al-Rikabi, Hanady Al-Shmgani, Yaser Hassan Dewir, Salah El-Hendawy

**Affiliations:** 1 Biology Department, College of Education for Pure Science/Ibn al-Haitham, University of Baghdad, Baghdad 10071, Iraq; rasha_fadhil@yahoo.com; 2 Plant Production Department, College of Food and Agriculture Sciences, King Saud University, Riyadh 11451, Saudi Arabia; shendawy@yahoo.com; 3 Department of Horticulture, Faculty of Agriculture, Kafrelsheikh University, Kafr El-Sheikh 33516, Egypt; 4 Agronomy Department, Faculty of Agriculture, Suez Canal University, Ismailia 41522, Egypt

**Keywords:** Hesperidin-inflammation, hesperidin-antioxidant, lipopolysaccharide, MCF7 apoptosis

## Abstract

(1) Background: Plant flavonoids are efficient in preventing and treating various diseases. This study aimed to evaluate the ability of hesperidin, a flavonoid found in citrus fruits, in inhibiting lipopolysaccharide (LPS) induced inflammation, which induced lethal toxicity in vivo, and to evaluate its importance as an antitumor agent in breast cancer. The in vivo experiments revealed the protective effects of hesperidin against the negative LPS effects on the liver and spleen of male mice. (2) Methods: In the liver, the antioxidant activity was measured by estimating the concentration of glutathione (GSH) and catalase (CAT), whereas in spleen, the concentration of cytokines including IL-33 and TNF-α was measured. The in vitro experiments including MTT assay, clonogenity test, and sulforhodamine 101 stain with DAPI (4′, 6-diamidino-2-phenylindole) were used to assess the morphological apoptosis in breast cancer cells. (3) Results: The results of this study revealed a significant increase in the IL-33 and TNF-α cytokine levels in LPS challenged mice along with a considerable elevation in glutathione (GSH); moreover, the catalase (CAT) level was higher compared to that of the control group. Cytotoxicity of the MCF-7 cell line revealed significant differences among the groups treated with different concentrations when compared to the control groups, in a concentration-dependent manner. Hesperidin significantly inhibited the colony formation of MCF7 cells when compared to that of control. Clear changes were observed in the cell shape, including cell shrinkage and chromatin condensation, which were associated with a later apoptotic stage. (4) Conclusion: The results indicate that hesperidin might be a potential candidate in preventing diseases.

## 1. Introduction

Flavonoids are groups of natural polyphenolic structures with diverse activities [1]. Hesperidin (HSP; C_28_H_34_O_15_), an active flavonoid abundantly found in citrus fruit, has various biological properties. In HSP structure, the aglycon (hesperetin or methyl eriodictyol) is bonded to rutinose [6-O-(α-l-Rhamnopyranosyl)-D-glucopyranose] and/or [6-O-(α-l-Rhamnosyl)-D-glucose], as a disaccharide [2]. Hydrolysis, demethoxylation, dehydration, dehydrogenation, demethylation, glucuronide binding, sulfate binding and N-acetylcysteine binding are considered the main types of HSP metabolism in rats [3]. Oxidative stress can occur as a consequence of imbalance between free radical and antioxidant in the cell, where proteins, lipids, DNA and other important molecules are damaged [4]. The strong relationship between inflammation and tumor and cancer progression has been reported [5]. The free radical scavenging activity by natural flavonoids is attributed to hydrogen donation from hydroxyl groups. HSP acts as an antioxidant and has free hydroxyl groups that donate electrons to free radicals [6]. The in vitro and in vivo studies reported that HSP possesses antihypertensive, hypolipidemic, anti-inflammatory, and anticarcinogenic activities [7,8], and is widely used in different industries and cosmetic products [9]. Literature reported that the possible mechanism behind the antioxidant activity of HSP and its derivatives might be due to the increased antioxidant cellular defenses via the ERK/Nrf2 signaling pathway, along with its radical scavenging activity; whereas, the anti-inflammatory properties reducing the inflammatory targets including NF-κB, iNOS, and COX-2, and the chronic inflammation markers [8,10]. The in vivo and in vitro study by Yeh et al., [11] reported that HSP suppressed the expression of TNF-α, IL-1β, IL-6, KC, MIP-2, MCP-1, and IL-12 and enhanced the production of IL-4 and IL-10 in mice lung lavage challenged with LPS. Interleukin-33 (IL-33), a member of IL-1 family, plays a vital role in different inflammatory diseases and cancer [12,13]. Blocking IL-33 could be a potential therapy that allow reducing cytokine-mediated malignant stem cell [13] as well as drug resistance [14].

Lipopolysaccharide (LPS) is commonly found in the cell wall of Gram-negative bacteria. It has been found that LPS stimulates the release and expression of inflammatory cytokines leading to an acute inflammatory response by activating the Toll-like receptor 4 (TLR4)-dependent pathway [15]. Binding of LPS to TLR4 activates the NF-κB and AP-1 signal transduction pathways via recruitment and activation of myeloid differentiation factor 88 expression, IL-1R kinase, TNFR associated factor 6, and NADPH oxidase [16]. The improved effects of HSP on reducing hepatotoxicity effect in rats induced by LPS administration has been reported [17]. Therefore, substances capable of inhibiting endotoxin inflammation have recently gained immense attention. The present study aimed to determine the potential protective role of HSP against LPS-induced oxidative stress and inflammation using BALB/c mice in the in vivo and in vitro experiments involving MCF7 breast cancer cell line.

## 2. Results

### 2.1. Free Radical Scavenging Activity

**Figure 1:**
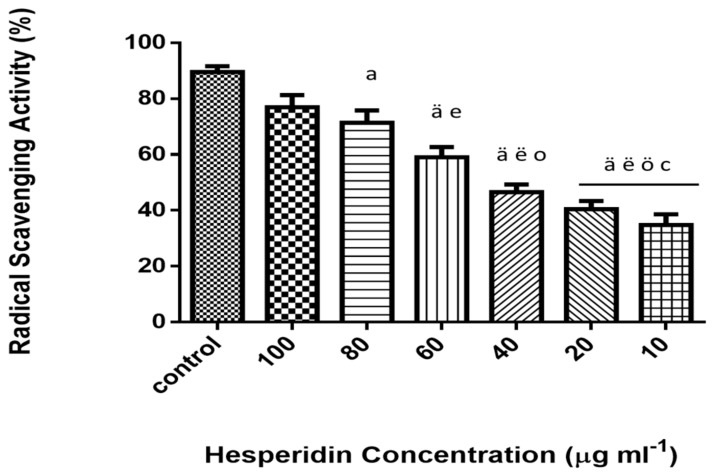
DPPH free radical scavenging activity of hesperidin (%) at different concentrations (10–100 µg mL^−1^). Data represent the means ± standard error (SE), n = 10. Control (vitamin c). ^a^ Significant difference from the control group at *p* < 0.05, ^ä^ at *p* < 0.001. ^e^ Significant difference from group treated with 100 µg mL^−1^ at *p* < 0.05, ^ë^ at *p* < 0.001. ^o^ Significant difference from group treated with 80 µg mL^−1^ at *p* < 0.05, ^ö^ at *p* < 0.01. ^c^ Significant difference from group treated with 60 µg mL^−1^ at *p* < 0.05 (IC50 value 53.46 µg ml^−1^).

2, 2-diphenyl-1-picrylhydrazyl (DPPH) is a free radical, which is stable at room temperature. It produces deep violet solution in organic solvents. DPPH is reduced in the presence of HSP molecules due to the presence of phenolic hydroxyl groups, resulting in decreased intensity of the colored solution. As presented in Figure 1, the antioxidant activities of HSP were monitored by using different concentrations. The results demonstrated an increased free radical scavenging activity in higher concentration along with concentration-dependent inhibition.

### 2.2. Viability and Cytotoxicity Assay

**Figure 2:**
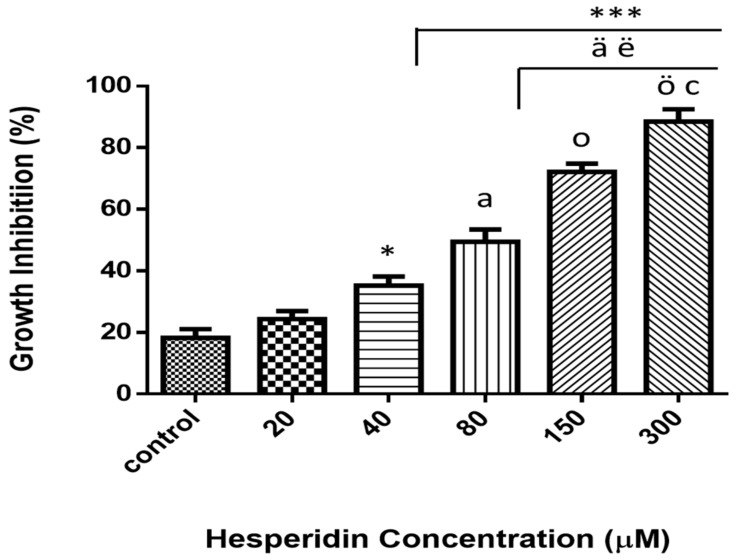
Cytotoxicity effect of various concentrations (0–300 µM) of hesperidin on MCF-7 breast cancer cell lines. Dose-dependent cytotoxicity effect of hesperidin was presented. Cells did not reveal significant cytotoxicity with lower dose at 24 h of exposure. * Significant differences versus control at *p* < 0.05, ***at *p* < 0.001. ^a^ Significant differences versus 20 µM at *p* < 0.01, ^ä^ at *p* < 0.001. ^ë^ Significant differences versus 40 µM at *p* < 0.01. ^o^ significant differences versus 80 µM at *p* < 0.01, ^ö^ at *p* < 0.001. ^c^ Significant differences between 150 NS 300 µM at *p* < 0.05. Data represent mean ± SD of three independent experiments, each comprising duplicate cultures.

Viability was examined via MTT colorimetric assay of MCF-7 cancer cell lines after 24 h of exposure at various concentrations (Figure 2). Different concentrations were used and ranging from 20 to 300 µM. The results illustrated that the treatment with HSP significantly inhibited the cell growth (*p* ≤ 0.05) and that the reduction was concentration-dependent.

### 2.3. Clonogenity Assay

**Figure 3:**
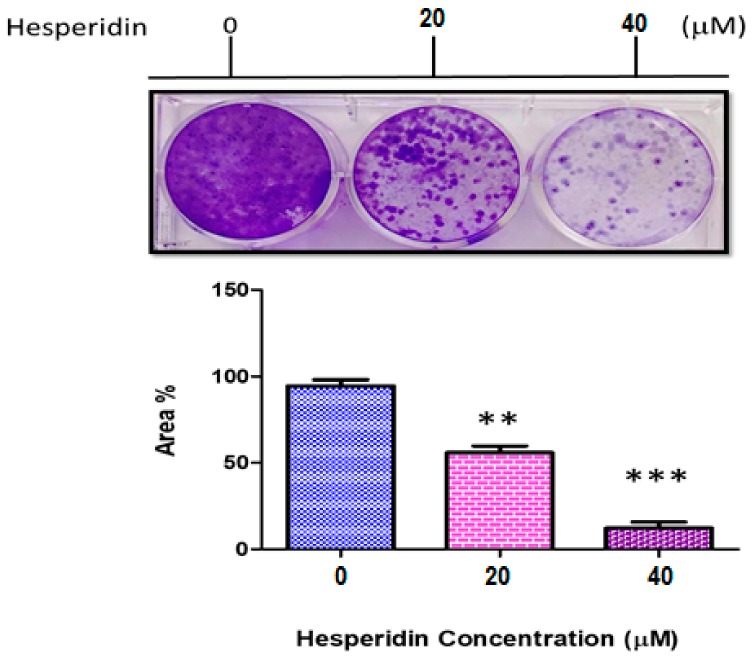
Clonogenity assay of MCF-7 cancer cell line not treated (0), 20, and 40 µM hesperidin (HSP). ** Significant differences versus control at *p* < 0.01, *** at *p* < 0.001. Data represent mean ± SD of three independent experiments, each comprising duplicate cultures.

The result of clonogenity assay is presented in Figure 3. The reduced colony count of MCF-7 was highly significant in both concentrations used compared to that of the control group. The reduced clonogenic activity was measured by using Image-J software. Significant results (*p* ≤ 0.001) were observed at concentration of 40 µM than those at 20 µM.

### 2.4. Apoptosis and Morphological Study using Sulforhodamine Staining

**Figure 4:**
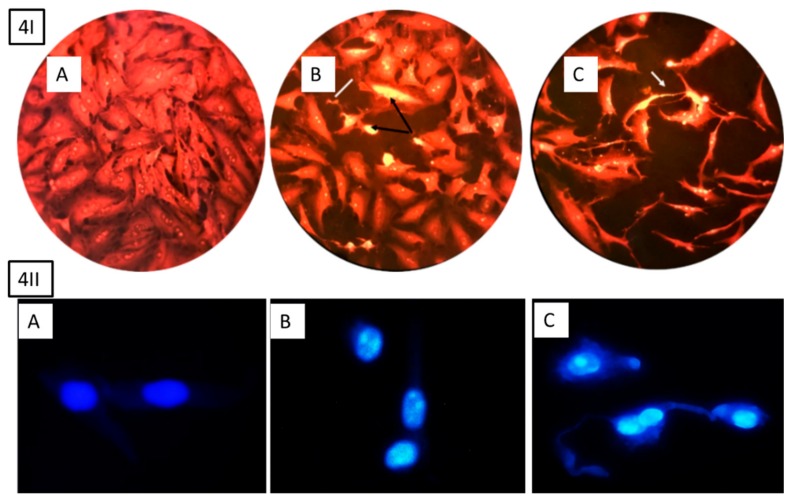
Morphological apoptosis of MCF-7 cells after treatment with hesperidin, 4I stained with sulforhodamine 101 and 4II cells stained with DAPI (20 and 40 µg ml^−1^). (**A**) Control cell has normal angular or polygonal shape with tact nucleus and is stained with a less bright fluorescence. (**B**) Treated cells with 20 µg ml^−1^ depict shrunken and condensed cytoplasm, chromatin (black arrow 4I), fragmentation, and apoptotic bodies (white line 4I). (**C**) Treated cells with 40 µg ml^−1^ clearly indicate late apoptosis including high cytoplasm and chromatin condensation, reduced cell count, and echinoid spikes on the surface of apoptotic cell (white arrow 4I).

To evaluate whether the cytotoxic effects were related to apoptosis or necrosis, the morphological changes were examined after treating the cells with HSP, as illustrated in Figure 4. The results indicated clear changes in the cell shape, which was associated with a later stage of apoptosis, including increased nuclear condensation and formation of apoptotic features.

### 2.5. Determination of Cytokine Levels

Table 1 and Table 2 present the hesperidin impact on IL-33 and TNF-α levels of spleen tissue in normal and LPS-induced inflammation mice. The results indicate that inflammation increased the cytokine secretion, as the levels of the both interleukins were significantly elevated (*P* < 0.05) in the LPS challenge group compared with the control group. In contrast, HSP treated mice presented an ameliorated anti-inflammatory reaction resulting in significantly decreased levels of IL-33 and TNF-α in mice that were cotreated with HSP and LPS; however, no significant effect was observed in the IL-33 and TNF-α levels in the mice injected with only HSP when compared to the control and cotreated mice.

### 2.6. Determination of Catalase and Glutathione

**Figure 5:**
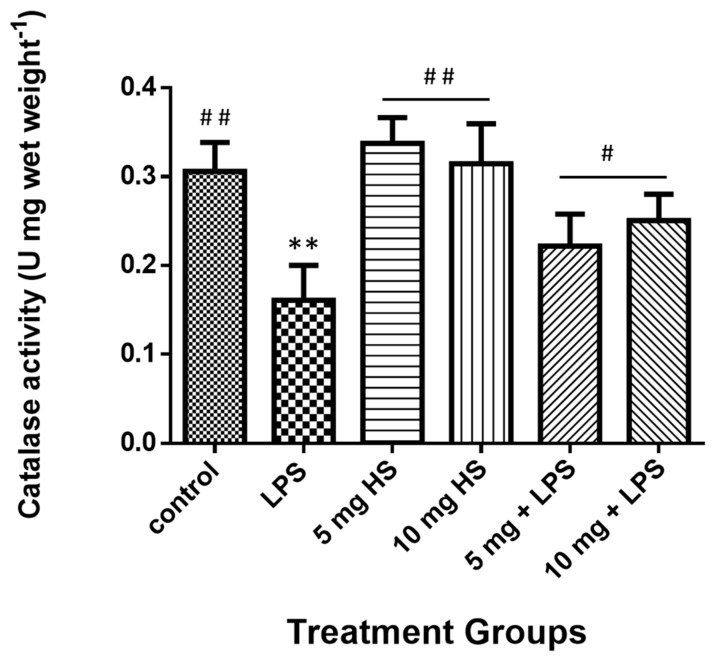
Treatment effects on the catalase activity in mice liver homogenate. Data represents the means ± standard error (SE), n = 10. ** Significantly different from control group at (*p* ≤ 0.01); # Significantly different from LPS group at (*p* ≤ 0.05), ^##^ at (*p* ≤ 0.01).

The liver total catalase level results (Figure 5) were significantly reduced in mice treated with LPS, whereas they significantly increased in the HSP-treated and cotreated groups. Moreover, no significant differences were observed between the HSP and control groups.

**Figure 6:**
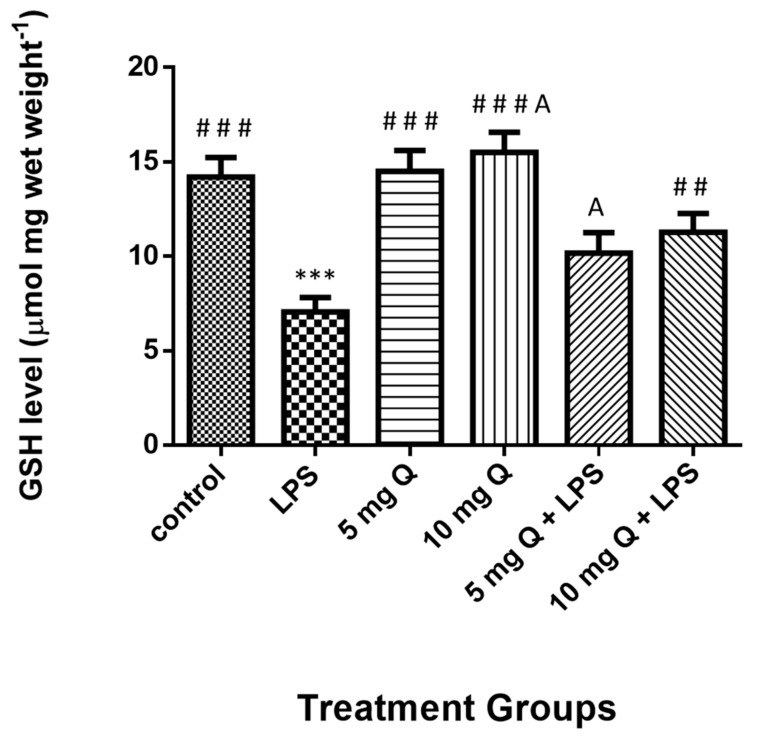
Treatment effects on the levels of glutathione level in mice liver homogenate. Data represent the means ± standard error (SE), n = 10. * Significantly different from the control group; # Significantly different from the LPS group, A significant difference between hesperidin 10 mg versus hesperidin (5 mg + LPS).

The liver total glutathione level results (Figure 6) were significantly reduced in mice treated with LPS, whereas they significantly increased in HSP-treated and co-treated groups. Although no significant differences were observed between the HSP and control groups, significant differences existed between the group treated with 10 mg of HSP only and that with 5 mg HSP and LPS.

## 3. Discussion

The present study evaluated the protective effects of HSP against the potential toxic effects of LPS on male mice, by which the oxidative stress and inflammations were investigated. Oxidative stress represents imbalance between free radicals and antioxidants in the cells. Raetz and Whitfield [18] reported that oxidative stress occurs due to an abnormal production of free radicals ROS, which is one of the hepatotoxic agents caused by LPS. The increase in the production of free radicals causing fat oxidation loses cell membranes, which is an important cause of destruction and damage to the cell membrane [19]. Our previous study [20], demonstrated that the liver is a target of LPS oxidative damage, which led to a significant increase in malondialdehyed (MDA) in the homogenate mice liver as a marker of lipid peroxidation. Similarly, Kaur et al. [17] reported the improvement of hesperidin in (LPS) endotoxin-induced hepatotoxicity and oxidative stress in the liver of rats; suggested mechanisms by inhibition of cytotoxic effect of nitric oxide and free radicals ROS in particular.. The results of present study clearly demonstrated that LPS administration significantly decreased the level of GHS and CAT. In contrast, HSP treatment revealed tremendous reduction in the lipid peroxidation and increased the antioxidant system capacity, wherein the level of CAT enzyme and GSH significantly increased in the mice cotreated with LPS and HSP. These results confirm the finding that HSP has a strong free radical scavenging potential as observed in the DPPH assay results. Flavonoids contain hydroxyl groups, specifically those comprising O-dihydroxy in ring B that may play a pivotal role in root inhibition. They provide greater stability and are involved in the transport of electrons between the hydroxyl groups of ring B [21]. Therefore, flavonoids and polyphenols act potent inhibitors of free radicals due to the high interaction of their hydroxyl substitutes [22]. Few studies demonstrated that HSP protected the organ tissues against various toxic agents, which induced oxidative damage such as TCDD, cadmium, and dimethylbenzanthracene [23,24]. Different mechanisms have been suggested for the beneficial effects of HSP against LPS damage including its antioxidant ability or suppression of AhR receptors in the cells [25]. 

The proinflammatory cytokines investigated in this study are part of an important mediator of inflammation response presenting cellular activity such as cell proliferation and apoptosis [26]. In this study, the cytokines levels were significantly increased in the spleen tissue after treatment with LPS; however, HSP treatment significantly decreased the levels of interested cytokines and consequently reduced the inflammation effects. Previous studies [27,28] reported the activated role of LPS in increased production of ROS from neutrophils and macrophages, consciously, increased the production of diverse inflammatory mediators which recruit more neutrophils to the tissue where inflammation process propagation. Therefore, it can be suggested that the protective effects of HSP moderated the inflammatory response along with antioxidant status against LPS-induced cytotoxicity in mice. In addition, it has been demonstrated that flavonoids inhibit the expression of proinflammatory genes in response to mediators like TNF-α and IL-β1 [29,30]. Thus, it can be suggested that the protective effect of HSP against LPS toxicity might be due to the decrease in the inflammatory cytokine levels. The protective effect of HSP against LPS toxicity might be due to the decrease in the inflammatory cytokine levels by the reduction in cytokines including TNF-α levels in stressed hepatocyte by which diminish significantly cell damages induced by LPS. 

## 4. Materials and Methods

### 4.1. Chemicals and Reagents

Hesperidin (≥80%), dimethyl sulfoxide (DMSO), and LPS (serotype E. Coli 0111:B4) liquid in PBS, (pH 7.4), DAPI (4′, 6-diamidino-2-phenylindole) and sulforhodamine 101 were obtained from Sigma Aldrich Co. (St. Louis, MO, USA). Cytokine kits (IL-33 and TNF-α) were purchased from Diaclone (Besancon Cedex, France). All other tissue culture chemicals and RPMI-1640 media of analytical grade were purchased from GIBCO (Rockville, MD, USA).

### 4.2. Animals and Experimental Design

Male mice BALB/c (20–25 g) were obtained and housed in the Animal House of Biological Department, College of Education for Pure Sciences/Ibn Al-Haitham, under controlled environmental conditions (12 light:12 dark light cycles; 25 ± 2 °C temperature). Water and food were provided *ad libitum*. Animals were categorized into six groups, each comprising 10 mice, and were injected intraperitoneally (i.p.) with HSP for 4 days as following: Group I (negative control) received 100 µl DMSO; Group II received 100 µl LPS; Group III was injected with 5 mg kg^−1^ HSP; Group IV was administered 10 mg/kg HSP. Groups V and VI received 5 and 10 mg kg^−1^ HSP, respectively, followed by intraperitoneal injection with LPS (50 µg ml^−1^) for 90 min.

### 4.3. Hesperidin Antioxidant Activity Measurement

The antioxidant activity was determined by using DPPH according to Al-Shmgani et al. [31]. Briefly, 1 mL of the samples was mixed with an equal volume of the DPPH solution (60 µM). After 30 min incubation at 37 °C in darkness, the absorbance was recorded at 517 nm spectrophotometrically (Perkin–Elmer Lambda 25, Germany). L-ascorbic acid was used as a positive control, and the measurements were carried out in triplicate. Inhibition of free radicals by DPPH was calculated by the following equation:

DPPH scavenging activity (%) = Ac − As/Ac × 100(1)

where, Ac = control absorbance and As = sample absorbance.

### 4.4. Determination of Antioxidants

Glutathione and catalase were determined in the liver homogenate according to the method by Sedlak and Lindsay [32]. The tissue was homogenized in PBS buffer at a ratio of 1 g tissue to 4 mL buffer, followed by centrifugation at 3000 rpm for 10 min at 4 °C. The supernatant was mixed with DTNB at 1:1 ratio. The absorbance was recorded at 415 nm, and the results were calculated by the following equation:

Glutathione activity (µmol mg wet weight^−1^) = (AB/E) ∗ L/mg liver weight (2)

AB = sample absorbance, E 1360, L = light length.

### 4.5. Determination of Catalase

Catalase activity was determined according to Huo et al. [33]. Briefly, 100 µl of samples was added to 1.9 mL of phosphate buffer; thereafter, 1 mL of H_2_O_2_ was added to all samples and the absorbance was read at 240 nm for 3 min.

### 4.6. Determination of Cytokines

Enzyme-linked immunosorbent assay (ELISA) was used to determinate the proinflammatory cytokines (Interleukin-33 (IL-33) and TNF-α) from spleen homogenate by using the available commercial kits. The assay was carried out according to the manufacturer’s instructions.

### 4.7. Cell Culture 

The MCF7 breast cancer cell line was provided by the Center of Biotechnology at AL-Nahrain University. RPMI-1640 medium containing 10% fetal bovine serum (FBS), 1% antibiotic (containing 10,000 U ml^−1^ penicillin G, 10 mg ml^−1^ streptomycin, and 25 µg ml^−1^ amphotericin B) was used in the cell culture and for maintenance. Cells were incubated at 37 ℃ in humidified 5% CO_2_ and were cultured at a concentration of 2.5 × 10^5^ cells ml^−1^ [34].

### 4.8. Measurement of Cell Viability

The colorimetric cell viability 3-(4,5-dimethylthiazol-z-yl)-2,5-diphenyltetrazolium (MTT) assay was used, as described by Berridge et al. [35]. Cells were seeded in 96-well plates at a concentration of 1 × 10^5^ cell ml^−1^; thereafter, HSP was added at concentrations of 10, 20, 40, 60, 80, and 100 µg ml^−1^. Culture in the medium served only as a control. The absorbance was measured at 620 nm using an enzyme-linked immunosorbent assay (ELISA) reader (VersaMaxTM, Molecular Devices, Sunnyvale, CA). Percentage of the inhibition ratio was calculated according to the following formula:

GI% = ((*OD of control wells* − *OD of control test wells*)/*OD of control wells*) × 100 (3)

where GI = growth index and OD = optical density.

### 4.9. Clonogenity Assay

The clonogenic cells were assayed according to the method of Liu, with certain modifications [36]. The cells were plated at a density of 10^4^ cells/well in 24 multiwell culture plates, and were then treated with 20 and 40 µM of HSP. After 24 h of treatment, the surviving cells were trypsinized, enumerated, and plated in Petri dishes at 5 × 10^3^ cells/well with a fresh medium. Cells colonies were fixed, stained with crystal violet for 30 min, and were then washed with tap water. Cells were then imaged with a digital camera and the clonogenic cells were determined as those able to form a colony with at least 50 cells. 

### 4.10. Morphological Evaluation of Apoptotic Cells

The combine of DAPI with sulforhodamine SR 101 was used [37] to evaluate the apoptotic features of cells. Fluorescent microscopy was used for this purpose (excitation/emission 358/461 nm for DAPI; 586/605 nm for sulforhodamine 101). MCF-7 cells were cultured in 96 multiwell plates at a density of 1 × 10^4^. After confluence monolayer, the cells were exposed to 20 and 40 µg ml^−1^ hesperidin for 24 h, and were then incubated at 37 °C with 5% CO_2_. Control cells were treated only with the media. Post-incubation, the media were removed, and the cells were washed twice with PBS. For fixative 1 mL of 96% ethanol for 30 min was added and left to air-dry at 25 °C. Stained with sulforhadamine 101-DAPI solution (prepared by mixing equal volumes of Sulforhodamine101 and DAPI solutions) was added for 30 min and left to air-dried. Then washed with PBS and covered with a cover-slip. The cells were examined immediately under a fluorescence microscope at 100× magnification power.

### 4.11. Statistical Analysis

Statistical analysis was performed using SPSS software version 16.0. All results were presented as means ± standard error. Significance was calculated using one-way analysis of variance (ANOVA), followed by Tukey’s test. A value of *p* < 0.05 was considered as statistically significant. 

## 5. Conclusions

In conclusion, the present study determined the oxidative stress and immune-toxic effects of LPS. The use of HSP reduced these negative effects by decreased levels of TNF-α and IL-β1 in the spleen tissue, and increased the antioxidant levels including CAT and GSH along with anticancer properties. Hence, HSP can be a promising potential medicinal agent.

## Figures and Tables

**Table 1:** IL-33 level in spleen homogenate of mice injected hesperidin for five days and treated with or without lipopolysaccharide (LPS). Experiment was performed in duplicate.

Treatment Groups	Dose Concentration	IL-33 Level (Mean ± SE) (pgww^−1^)
Control (DMSO + PBS)	5 µg ml^−1^	40.582 ± 8.043 ^#^
LPS only	5 µg ml^−1^	225.13 ± 13.20 *
Hesperidin only	5 µg kg^−1^	43.78 ± 14.72 ^#^
Hesperidin only	10 µg kg^−1^	68.22 ± 19.40 ^# NS^
Hesperidin + LPS	5 µg kg^−1^ + 5 µg ml^−1^	129.18 ± 18.03 *^#^^a^
Hesperidin + LPS	10 µg kg^−1^ + 5 µg ml^−1^	102.09 ± 14.20 *^#^^a^

(*) indicate significant differences at *p* < 0.05 versus control, (#) indicate significant differences at *p* < 0.05 versus LPS, (a) Significant (*p* < 0.05) between hesperidin versus hesperidin with LPS, (NS) not significant, (*p* > 0.05) between same hesperidin treatment groups with different concentration, (ww) wet weight.

**Table 2:** TNF-α level in spleen homogenate of mice injected hesperidin for five days and treated with or without LPS. Experiment was performed in duplicate.

Treatment Groups	Dose Concentration	TNF-α Level (pg mg ww^1^)
Control (DMSO + PBS)	5 µg ml^−1^	119.56 ± 9.81 ^#^
LPS only	5 µg ml^−1^	338.86 ± 22.87 *
Hesperidin only	5 µg kg^−1^	256.1 ± 23.61 ^#^
Hesperidin only	10 µg kg^−1^	230.75 ± 15.39 ^#^^NS^
Hesperidin + LPS	5 µg kg^−1^ + 5 µg ml^−1^	285.55 ± 22.27 *^#^
Hesperidin + LPS	µg kg^−1^ + 5 µg ml^−1^	267.73 ± 13.27 *^#^^NS^

(*) indicate significant differences at *p* < 0.05 with control, (#) indicate significant differences at *p* < 0.05 with LPS, (NS) not significant (*p* > 0.05) between same hesperidin treatment groups with different concentration, (ww) wet weight.
